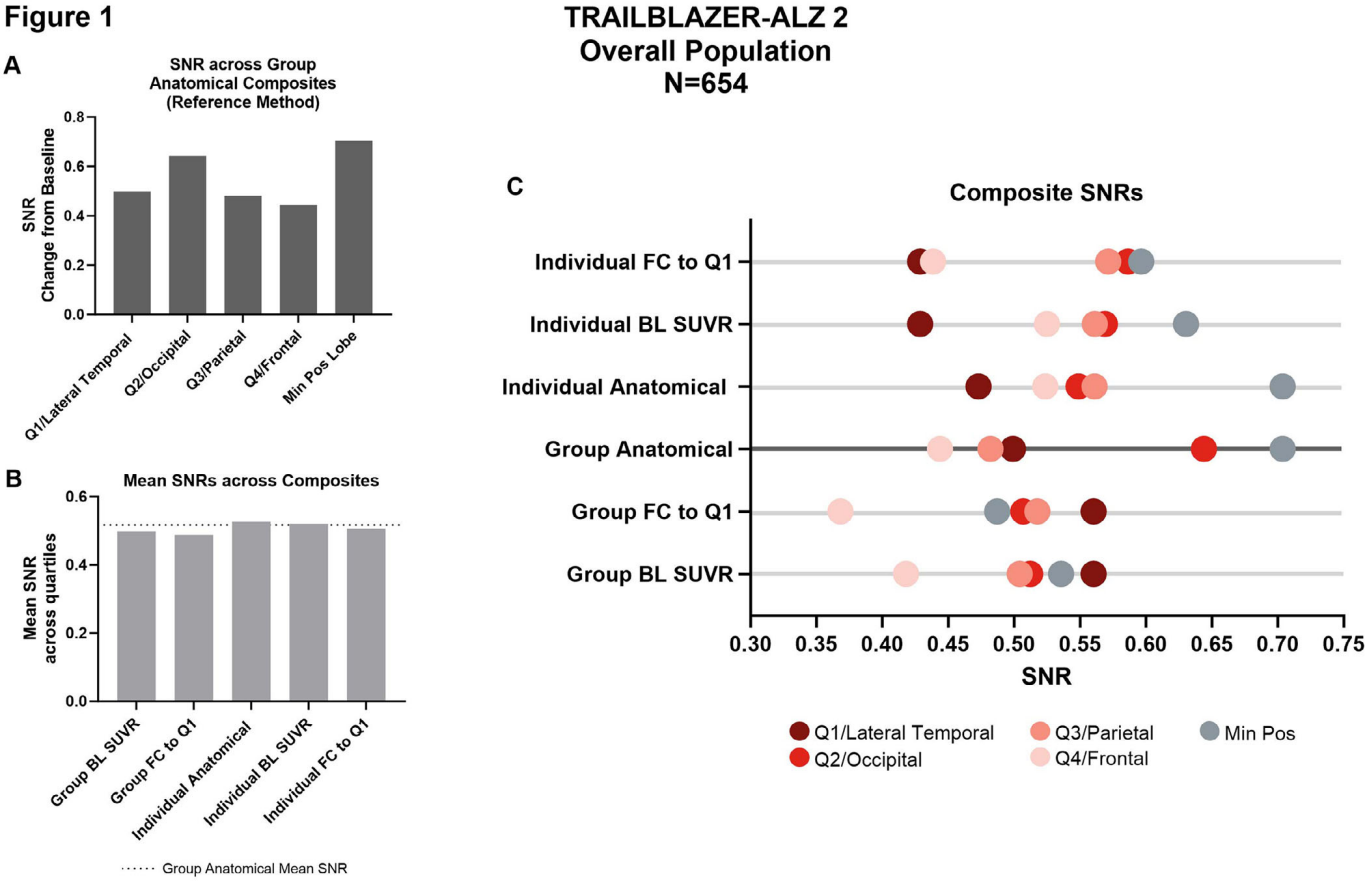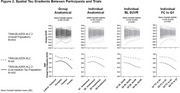# Regional tau quantification methodologies in early symptomatic participants with presence of tau pathology

**DOI:** 10.1002/alz70856_104873

**Published:** 2026-01-07

**Authors:** Diana O Svaldi, Leonardo Iaccarino, Ixavier A. Higgins, Vikas Kotari, Nicolai Franzmeier, Michael Ewers, Sergey Shcherbinin

**Affiliations:** ^1^ Eli Lilly and Company, Indianapolis, IN, USA; ^2^ Institute for Stroke and Dementia Research (ISD), LMU University Hospital, LMU, Munich, Bavaria, Germany; ^3^ Institute for Stroke and Dementia Research (ISD), LMU University Hospital, LMU Munich, Munich, Germany

## Abstract

**Background:**

This study compares approaches for defining target regions in their ability to consistently capture tau progression relative to anatomical composites used in Alzheimer's disease trials.

**Method:**

Paired, baseline and 18‐month regional flortaucipir data were included from TRAILBLAZER‐ALZ2 (NCT04437511, Low/Medium/High Tau) and TRAILBLAZER‐ALZ (NCT03367403, Low/Medium Tau) placebo participants. Regional SUVRs were combined into four composite regions according to individual‐ versus group‐level and anatomical‐ versus data‐driven approaches. Composites were defined and ranked according to hypothetical longitudinal spatial gradients based on descending mean regional SUVR (Q1→Q4). Anatomical composites were defined by lobes, whereas data‐driven composites (quartiles) were defined by either baseline SUVR or functional connectivity (FC) to the highest baseline SUVR quartile. Group‐level methodologies used mean regional SUVRs across participants, whereas individual‐level used single participant regional SUVRs. Finally, the minimum positive composite (lowest SUVR>2 standard deviations (SD) above amyloid‐negative cohort mean) was considered a target region. Longitudinal signal‐to‐noise (SNR) for each composite (mean/SD of change) and mean SNR across composites were compared to group lobar composites (reference method, Figure 1A). Homogeneity between individual‐level spatial gradients and across trial‐level spatial gradients were evaluated for methods showing similar/superior SNR to reference.

**Result:**

Group‐level, data driven approaches exhibited lower mean SNR across composites relative to the reference (Figure 1). Individualized methods exhibited similar/higher mean SNR (Figure 1). Data‐driven individualized methods also exhibited significantly increased spatial homogeneity (*p* < 0.05 Mann Kendall test) between participants (Figure 2) and visually across trials (Figure 2) versus the reference. Minimum positive composites exhibited superior SNR relative to Q1‐Q4 across individualized and anatomical methodologies (Figure 1C). Spatial gradients were similar between SUVR‐based and FC‐based methodologies (Figures 1‐2).

**Conclusion:**

Participant specific target regions may improve consistency in longitudinal tau PET analyses. For each participant, focusing on regions that have recently reached positivity may improve SNR. The similarity between SUVR‐based and FC‐based methods continues to suggest connectivity between regions is a significant mechanism contributing to tau spread. Additional analyses to support results summarized here as well as analyses testing these methodologies on asymptomatic individuals from A4 may also be presented.